# Anti-Angiogenic Effects of Natural Compounds in Diet-Associated Hepatic Inflammation

**DOI:** 10.3390/nu15122748

**Published:** 2023-06-14

**Authors:** Sara Novi, Vincenzo Vestuto, Pietro Campiglia, Nicola Tecce, Alessia Bertamino, Mario Felice Tecce

**Affiliations:** 1 Department of Pharmacy, University of Salerno, Via G. Paolo II, 84084 Fisciano, Italy; snovi@unisa.it (S.N.); vvestuto@unisa.it (V.V.); pcampiglia@unisa.it (P.C.); abertamino@unisa.it (A.B.); 2 Unit of Endocrinology, Department of Clinical Medicine and Surgery, Medical School of Naples, Federico II University, Via Sergio Pansini 5, 80131 Napoli, Italy; nicola.tecce@unina.it

**Keywords:** liver disease, inflammation, steatosis, ALD, NAFLD, ASH, NASH, angiogenesis, hepatocellular carcinoma, natural compounds

## Abstract

Alcoholic liver disease (ALD) and non-alcoholic fatty liver disease (NAFLD) are the most common causes of chronic liver disease and are increasingly emerging as a global health problem. Such disorders can lead to liver damage, resulting in the release of pro-inflammatory cytokines and the activation of infiltrating immune cells. These are some of the common features of ALD progression in ASH (alcoholic steatohepatitis) and NAFLD to NASH (non-alcoholic steatohepatitis). Hepatic steatosis, followed by fibrosis, lead to a continuous progression accompanied by angiogenesis. This process creates hypoxia, which activates vascular factors, initiating pathological angiogenesis and further fibrosis. This forms a vicious cycle of ongoing damage and progression. This condition further exacerbates liver injury and may contribute to the development of comorbidities, such as metabolic syndrome as well as hepatocellular carcinoma. Increasing evidence suggests that anti-angiogenic therapy may have beneficial effects on these hepatic disorders and their exacerbation. Therefore, there is a great interest to deepen the knowledge of the molecular mechanisms of natural anti-angiogenic products that could both prevent and control liver diseases. In this review, we focus on the role of major natural anti-angiogenic compounds against steatohepatitis and determine their potential therapeutic benefits in the treatment of liver inflammation caused by an imbalanced diet.

## 1. The Liver: Function and Diseases

The liver is an essential organ that plays a critical role in maintaining the metabolic and biochemical balance of the body. It is involved in nutrient metabolism, the regulation of blood volume, the storage of vitamins and minerals, the support of the immune system, endocrine control of growth signaling, lipid and cholesterol homeostasis, and the degradation of xenobiotic compounds. In addition, its ability to oxidize lipids and package their excess for secretion and storage in other tissues makes it the major player in fat accumulation in various diseases [1].

The liver is composed of several cell types, mainly including hepatocytes, cholangiocytes, stellate cells, sinusoidal cells, and Kupffer cells. Hepatocytes are the most abundant cells in the liver volume, and they perform many biological functions attributed to this organ; cholangiocytes are polarized cells that line the bile ducts within the liver and play a crucial role in the secretion and modification of bile, which is essential for the digestion and absorption of fats in the small intestine, in addition to their immunological functions; hepatic stellate cells (HSCs) are a multifaceted cell population existing as a quiescent form that can be activated when damage is induced to promote wound healing; sinusoidal endothelial cells are a specialized endothelial population that coat the hepatic sinusoids and allow for the exchange of nutrients, hormones, and waste products between the blood and the liver cells; and finally, Kupffer cells are a type of dedicated macrophage of the liver that help to remove foreign particles such as bacteria and viruses [2].

Hence, the liver plays a vital role in the body, but it is also true that it is susceptible to various diseases and conditions that can impair its function and overall health. Liver diseases encompass a wide range of conditions caused by factors such as viral infections, alcohol abuse, genetic disorders, autoimmune responses, obesity, and certain medications. These diseases can range from mild and reversible to severe and chronic. Common liver diseases include hepatitis, which refers to liver inflammation caused by viruses, alcohol, drugs, or autoimmune disorders. Chronic hepatitis can lead to cirrhosis, liver failure, or liver cancer if untreated. Cirrhosis is characterized by the replacement of healthy liver tissue with scar tissue, often resulting from long-term alcohol abuse or chronic viral hepatitis. It can lead to liver failure, portal hypertension, and hepatocarcinoma (HCC).

In this broad scenario of chronic ailments, ALD (alcoholic liver disease) and NAFLD (non-alcoholic fatty liver disease) are the most common causes of liver disease, especially in Western countries [3,4].

### 1.1. ALD and NAFLD

In the industrial city, increasing alcohol abuse and excessive eating are responsible for ALD and NAFLD, respectively; both diseases have hepatic steatosis in common and range from a mild condition to more aggressive forms of the disease called alcoholic steatohepatitis (ASH) and non-alcoholic steatohepatitis (NASH), where steatosis, hepatocyte apoptosis, inflammation, and fibrogenesis coexist [5,6].

#### 1.1.1. ALD Pathogenesis

Hepatic steatosis represents the first step of these pathologies and is characterized by the accumulation of lipids, especially triglycerides, in the cytoplasm of the hepatocytes, and the absence of inflammation, cell death, or hepatic fibrosis. In this regard, the following two forms of steatosis can be distinguished: a macrovesicular form, where there is an arrangement of fat in a single large droplet that dislodges the nucleus in a peripheral location of the hepatocyte, and a microvesicular form, where fat collects in small droplets that do not dislodge the nucleus of the cells. These two aspects often reflect different stages of steatosis, and indeed, clinical cases in which the two forms are associated are not uncommon [7,8].

Many hypotheses were proposed to explain the pathogenetic mechanisms of ALD. As the liver is the main organ responsible for alcohol metabolism, ethanol and its metabolites (acetaldehyde-acetate, fatty acid ethanol esters (FAEEs), ethanol-protein adducts) may exert a direct cytotoxic effect as hepatotoxins [9].

The metabolism of ethanol in the liver involves both oxidative and non-oxidative pathways. The oxidative pathway is enabled by alcohol dehydrogenase (ADH) and acetaldehyde dehydrogenase (ALDH). ADH and ALDH convert ethanol to acetaldehyde and acetaldehyde to acetate, respectively [10]. The end products of these reactions are acetaldehyde, acetate, and large amounts of NADH. Acetaldehyde injures the liver by directly inducing inflammation, extracellular matrix deposition, and remodeling, leading to the development of fibrosis [11]. In addition, it covalently binds to biomolecules, prompting lipid peroxidation and the subsequent production of toxic metabolites (e.g., malondialdehyde, 4-hydroxy-2,3-trans-nonenal), which are commonly analyzed under oxidative stress conditions [12,13]. Finally, acetaldehyde stimulates transforming growth factor (TGF)-β signaling in HSCs, which acquire a pro-fibrogenic and pro-inflammatory profile [14].

An increase in the NADH/NAD^+^ ratio can affect the biochemical reactions in the mitochondria and the gene expression in the nucleus. In particular, the re-oxidation of NADH requires additional oxygen in the mitochondria. To this end, hepatocytes absorb more than their normal consumption of oxygen, but not enough to fulfill the needs of all the liver regions adequately. Therefore, alcohol consumption causes significant hypoxia in perivenous hepatocytes, which are the first to show evidence of chronic alcohol-induced damage [15].

**Figure 1:**
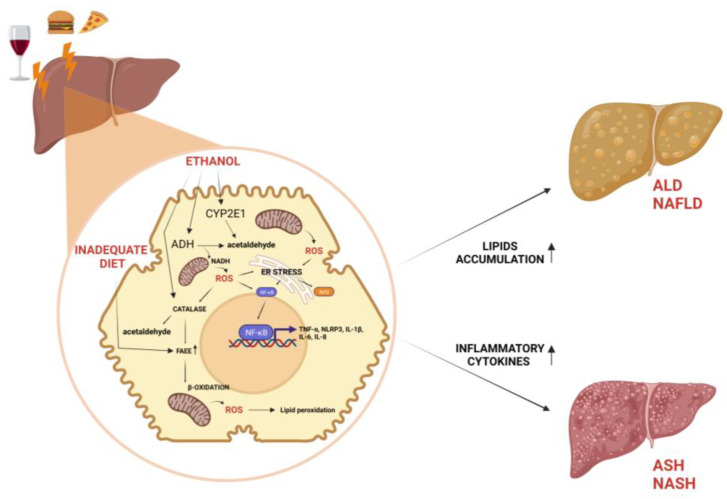
Role of alcohol consumption and inadequate diet on liver disease. ADH, catalase, and CYP2E1 contribute to oxidative metabolism of ethanol to generate acetaldehyde. Alcohol metabolism, high fat, and high carbohydrate diet induce NAFLD and ALD by means of FAEEs accumulating in lipid droplets. Accumulation of ROS resulting from ethanol or β-oxidation leads to formation of lipid peroxides and ER stress, which induce NF-κB activation and consequent inflammatory state at the base of ASH and NASH. ↑ = increased effect.

Cytochrome P450, in particular, cytochrome P450 2E1 (CYP2E1), is upregulated in chronic alcohol abuse and assists ADH in the conversion of alcohol to acetaldehyde [16] (Figure 1). Reactive oxygen species (ROS), such as hydrogen peroxide and superoxide ions, generated by CYP2E1, are responsible for the pro-inflammatory profile of alcohol-induced liver injury by the following:

(1) The activation of redox-sensitive transcription factors (e.g., nuclear factor kappa B (NF-κB), nuclear factor erythroid 2-related factor 2 (Nrf2), activating transcription factor 4 (ATF4), etc.) [17,18,19];
(2) The recruitment of neutrophils and other immune cells [20];
(3) Increasing levels of circulating inflammatory cytokines (e.g., tumor necrosis factor-α (TNF-α) and interleukin-1β (IL-1β)) [18];
(4) Exacerbated lipid peroxidation associated with alcoholic liver injury [21].

Finally, the last player in the inflammatory and fibrogenic phenomena is catalase, a peroxisomal enzyme involved in the regulation of non-oxidative alcohol metabolism, the product by which FAEEs are responsible for alcoholic steatosis and useful as biomarkers of chronic alcohol consumption [16] (Figure 1).

#### 1.1.2. NAFLD Pathogenesis

Obesity, type 2 diabetes, hyperlipidemia, and other conditions associated with insulin resistance are commonly present in patients with metabolic syndrome (MS). Multiple occurrences suggest that NAFLD is associated with MS. The latter is a clinical entity characterized by a cluster of metabolic changes, including glucose intolerance, dyslipidemia, and hypertension [22,23,24]. Approximately 90% of NAFLD patients have one or more diagnostic criteria for MS, allowing NAFLD to be defined as the “hepatic representation” of MS [25].

We can distinguish the following two types of NAFLD: primary (related with MS) and secondary (related with metabolic or iatrogenic conditions, distinct from MS). The pathophysiology of primary NAFLD is not fully understood.

Since 1998, the two-hit pathogenetic model of liver injury proposed by Day and James has been widely accepted [26]. The first hit leads to the initial accumulation of triglycerides within the hepatocyte, caused by an increased hepatic lipid uptake [27]. Later, the condition of steatosis thus predisposes the liver to the second event (second hit), after which inflammation, fibrosis, and liver damage develop. The factors determining the second hit include several biomolecules that are capable of interfering with the insulin-induced signal transduction mechanism, including inflammatory cytokines (e.g., TNF-α, IL-6, IL-1β, hormones produced by adipose tissue such as adipokines, leptins, and adiponectins), oxidative stress, lipid peroxidation markers, fatty acids, and even ceramides.

In fact, the inhibition of ceramide synthesis relieves NAFLD and fibrosis. Moreover, increased hepatic steatosis is associated with ceramide-rich hepatic lipids [28,29].

In addition, insulin resistance results in increased hyperglycemia-induced endoplasmic reticulum (ER) stress, peripheral lipolysis, hepatic fatty acid (FA) uptake, and hepatic synthesis of de novo lipids [30,31,32]. Therefore, the oxidation and disposal of fatty acids result in a defect compared to their accumulation and neo-synthesis, leading to an accumulation of fats within the hepatocytes with important consequences, such as dysregulated ketogenesis [33,34]. In addition, fatty acids negatively affect the intracellular insulin signaling mechanism and cause hepatic insulin resistance through multiple pathways mediating the activation of protein kinase C (PKC-3), c-Jun N-terminal kinase (JNK), I-kappaB (I-κB) kinase β, and NF-κB [35,36,37].

Furthermore, insulin resistance was found to be associated in increased mitochondrial fatty acid oxidation. This process, both mitochondrial and peroxisomal, can produce ROS that are hepatotoxic and contribute to the development of oxidative stress. In addition, fatty acids and their metabolites are ligands of PPARα (peroxisomal proliferator-activated receptor-α), a transcription factor that regulates the expression of several genes encoding enzymes involved in fatty acid oxidation at the mitochondrial, peroxisomal, and microsomal levels. In support of this, the deletion of PPARα in the hepatocytes impaired the fatty acid catabolism, resulting in hepatic lipid accumulation in the steatosis models [38,39].

Regarding the role of inflammatory cytokines in the pathogenesis of NAFLD, they could cause hepatic and systemic insulin resistance, as well as promote liver injury, apoptosis, neutrophil chemotaxis, and HSC activation [40,41,42]. Studies reported several cytokines involved in the genesis and progression of NAFLD, such as IL-1β, IL-6, TNF-α, C-reactive protein (CRP), and NOD-like receptor protein 3 (NLRP3) inflammasome activation. Some of these inflammatory products can be used as biomarkers to reveal the stage of NAFLD [43,44] (Figure 1).

In addition, fatty acids accumulated in the hepatocytes can stimulate cytokine production via the NF-κB-dependent pathway [45]. An additional source of pro-inflammatory cytokines is the infiltrating macrophages in the adipose tissue of obese subjects; cytokines are increased in addition to promoting the onset of a state of insulin resistance. They appear to reduce the production of certain peptides produced by visceral adipose tissue, such as leptins, resistins, and adiponectins, which are involved in the pathogenesis of NAFLD [46].

### 1.2. ASH and NASH

Alcoholic steatohepatitis (ASH) and non-alcoholic steatohepatitis (NASH) are globally prevalent forms of chronic liver disease. ASH develops due to long-term alcohol consumption, whereas NASH is linked to unhealthy dietary patterns, obesity, insulin resistance, and type 2 diabetes. [47]. ASH and NASH share common features despite their distinct causes. Both conditions involve the accumulation of excessive fat in the liver, which leads to lipotoxicity and triggers inflammation, causing hepatocellular swelling and promoting fibrosis development [48].

Between 90 and 100% of individuals who consume more than 40 g of alcohol per day will eventually develop alcoholic fatty liver. Approximately 10–35% of these individuals develop ASH, which is characterized by a severe inflammatory state of the liver, hepatocyte swelling, neutrophil infiltration, and/or hepatic fibrosis [49]. In approximately 8–20% of patients with ASH, the risk of developing HCC increases by approximately 2% per year [50]. Factors involved in the pathophysiology of ASH include steatosis, reactive oxygen species, acetaldehyde-mediated toxicity, and cytokine-induced inflammation, but hepatic inflammation is the key requirement to flow into liver fibrosis, cirrhosis, and HCC [51].

According to recent studies, a portion of patients with NAFLD progress to NASH over time. Approximately 5–20% of NAFLD patients develop NASH during their illness. Additionally, 10–20% of NAFLD patients experience a higher grade of fibrosis, indicating increased liver scarring. However, the progression to cirrhosis, which is severe liver fibrosis, occurs in less than 5% of NAFLD patients [52]. Thus, ASH and NASH share a common pathogenesis and are mediated by different mechanisms [53]. The “two-hit” theory suggests that oxidative stress and cytokines lead to the progression of necroinflammation, and ultimately, to fibrosis and cirrhosis [54]. Indeed, hepatic macrophages and Kupffer cells are able to activate HSCs through the production of IL-6 and TNF-β, while HSCs, in turn, produce pro-inflammatory cytokines, such as TNF-α and IL-8 [55]. Fat-derived products can also cause inflammation and injury in the liver by activating the inflammasome and inducing the production of IL-1 and IL-18. Fat products can also cause inflammation and injury in the liver by activating the NLRP3 inflammasome, which induces the production of IL-1β and IL-18; evidence for the critical role of NLRP3 activation is the marked protection from disease in knockout mice following acute or chronic alcohol administration [56]. In addition, oxidative stress strains antioxidant defenses, which become inadequate. This results in liver injury through direct cellular damage and NF-κB-mediated cellular signaling [57]. Several studies also show that oxidative stress and the increase in ER stress-mediated Nrf2 are induced, which appear to be some common features of ASH and NASH (Figure 1); accordingly, altered markers of ER stress are recorded in numerous experimental preclinical models whose diets are administered alcohol or HFD [58].

As previously reported, the mechanisms that drive disease progression also induce steatosis. Therefore, steatosis could be considered as an “adaptive” response of the liver to stress, and continuous insult leads to ASH and NASH, in which we find not only steatosis, but also inflammation and fibrosis [59,60].

In addition to oxidative stress and cytokine production, insulin resistance and hyperglycemia are active players in the progression of the pathology by directly inducing fibrosis or by regulating connective tissue synthesis [61,62].

Unfortunately, although significant advances were made in identifying the leading actors that mediate the transition from steatosis to steatohepatitis, the treatment options for ASH and NASH are restricted [63]. Exercise and dietary interventions remain the main recommendations for patients with NAFLD and NASH. However, it is difficult for patients to adhere to such lifestyle changes for a variety of social, psychological, physical, genetic, and epigenetic conditions; therefore, pharmacotherapy is essential [64,65].

## 2. Angiogenesis and Hepatic Inflammation

In this review, we focus on the role of the main natural compounds with health beneficial properties, including their role in angiogenesis within the pathophysiology of liver fibrosis.

Angiogenesis is a well-documented and extensively studied process that involves the formation of new blood vessels from pre-existing vasculature [66,67]. The angiogenic phenomenon occurs physiologically during normal tissue growth and wound healing, but also in pathological contexts such as liver disease and cancerogenicity. Although the formation of new blood vessels is an attempt to deliver oxygen and nutrients to the injured liver tissue, promoting tissue repair, on the other hand, an excessive and disorganized angiogenesis, can contribute to liver fibrosis, impair liver function, and create an environment favorable for the development of liver cancer [68,69].

In the liver, angiogenesis proceeds with molecular mechanisms like those demonstrated in other parts of the body. However, several differences in this organ make angiogenesis more complex, such as a more complex parenchyma with two distinct microvascular structures (portal vein and hepatic sinusoids), the presence of liver-derived angiopoietin-like peptide 3 (ANGPTL3), and the activity of HSCs, which play an active role in liver fibrosis by proliferating and becoming myofibroblasts that can modulate angiogenesis. Finally, the unique microenvironment of the liver compared to the other organs must be emphasized. Liver sinusoidal endothelial cells have fenestrations that allow for the direct contact between the hepatocytes and the blood. This close interaction influences the angiogenic process in the liver and can have implications for therapeutic targeting [70,71,72,73].

**Figure 2:**
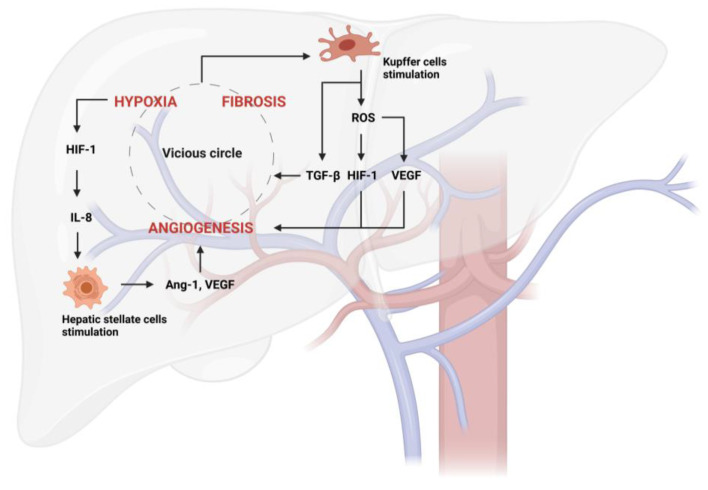
Vicious circle generated from hypoxia, fibrosis, and angiogenesis in liver disease. Hypoxia activates hypoxia-inducible factors (HIFs), such as HIF-1, which, among other things, stimulates hepatic stellate cell (HSC) activation via IL-8 production. They consequently produce angiogenic factors, such as Ang-1 (Angiopoietin-1) and VEGF (vascular endothelial growth factor). The stimulation of Kupffer cells by the vicious circle further aggravates this pathological process through TGF-β release. Furthermore, Kupffer cells can produce ROS-inducing new vessel formation through the stimulation of HIF-1 and VEGF.

Multiple factors trigger angiogenesis in liver disease, including tissue inflammation and fibrosis. These lead to progressive tissue hypoxia, which, in turn, causes the capillarization of sinusoids and the initiation of the angiogenic process that leads to the progression of NAFLD and ALD to NASH and ASH, respectively. Hypoxia activates angiogenesis through signaling mediated by transcription factors called hypoxia-inducible factors (HIFs), such as HIF-1 (Figure 2), which, among other things, stimulates inflammation via the NF-κB pathway [74].

All of this occurs because the intrahepatic vascular remodeling, which causes the deposition of fibrillar collagen (type I) instead of sinusoidal collagen (type IV), generates a continuous capillarization of the sinusoids and the development of intrahepatic shunts, leading to a decreased effective hepatocyte perfusion and the consequent loss of specific endothelial fenestrations. This process, together with the accumulation of fibrotic tissue, creates vascular resistance and decreases oxygen transport to the liver parenchyma, resulting in the upregulation of pro-angiogenic mechanisms via hypoxia, and establishing a vicious cycle [75,76,77].

In that context, liver angiogenesis primarily occurs as part of chronic liver diseases characterized by inflammation, fibrosis, and tissue damage. In contrast, angiogenesis in other organs can be triggered by various factors, such as wound healing, tumor growth, and tissue regeneration following injury or ischemia. Moreover, in pathological liver angiogenesis, there is a strong dialogue between different cell populations; indeed, wound healing generates an increase in the expression of some cytokines and factors with pro-angiogenic and inflammatory actions. Kupffer cells may contribute to angiogenesis by releasing ROS and inducing new vessel formation by stimulating the expression of TNF-α, HIF-1, and VEGF (vascular endothelial growth factor) [78]. Upon liver injury, these cells further produce TGF-β, the major regulator in chronic liver disease, contributing to all stages of disease progression from initial insult, through inflammation and fibrosis, to cirrhosis and HCC [79] (Figure 2). HSCs express several chemokines capable of stimulating angiogenesis and respond to hypoxia in a HIF-1α-related pathway through the production of IL-8 and the increase in VEGF, Ang-1 (angiopoietin-1), and their corresponding receptors VEGFR-2 and Tie-2 [80,81] (Figure 2). In addition, other cells that are not present in the liver are activated; leukocytes and mast cells are involved in the regulation of angiogenesis by releasing histamine, heparin, cytokines, and other angiogenic factors (Ang-2, EGF (epidermal growth factor), PlGF (placental growth factor), TGF-β1, VEGF, etc.) [82,83].

Therefore, angiogenesis represents a key stage of inflammation and fibrosis [84] in liver disease. For these reasons, the recognition of angiogenesis as a key process in chronic liver diseases has opened up possibilities for targeted therapeutic interventions. Several anti-angiogenic molecules (e.g., the anti-vascular endothelial growth factor (VEGF) monoclonal antibodies bevacizumab, brivanib, dovitinib, nintedanib, etc.) are currently used in the treatment of various cancers, including HCC, suggesting that angiogenesis is a promising therapeutic target [85,86,87]. Hence, it is of great importance to determine the potential therapeutic benefits of various natural compounds in the prevention and treatment of liver inflammation. In this scenario, there are several natural compounds that are active in this field.

## 3. Phytochemicals to Counteract the Stages of Liver Disease via Angiogenesis Inhibition

### 3.1. Quercetin

Quercetin is a flavonoid that is found in fruits such as apples, red grapes, citrus fruits, tomatoes, onions, and other green leafy vegetables. Quercetin possesses various biological and pharmacological activities, including antioxidant, antiviral, anti-inflammatory, antiproliferative, and antifibrotic effects [88,89]. In recent studies, quercetin inhibited hepatic inflammation and fibrosis in mice by downregulating the HMGB1-TLR2/4-NF-κB signaling pathway [90]. Moreover, it also limited hepatic fibrosis by inhibiting HSC activation and reducing autophagy by regulating crosstalk between the TGF-β1/Smads and PI3K/Akt pathways [91].

Nevertheless, recent data show that macrophages play a complex role in liver fibrogenesis and are directly involved in the progression and resolution of liver fibrosis [92,93]. Indeed, inflammatory cytokines released by macrophages perpetuate inflammation and activate HSCs; quercetin showed direct effects by reducing the number of hepatic macrophages and ameliorating liver fibrosis after treatment of mice with CCl_4_ [94] (Table 1). In conclusion, quercetin is a flavonoid with numerous biological benefits, and because of its anti-inflammatory and antifibrotic effects, it may hold promise as a potential therapeutic agent for liver fibrosis.

### 3.2. Silybin

Silybin is a flavonolignan that exists as a mixture of two diastereomers, silybin A and silybin B, in a roughly equimolar ratio [95]. It is usually extracted together with other compounds from milk thistle (*Silybum marianum*) in a mixture called silymarin, consisting of 50–60% silybin, with the rest consisting of silydianin, silycristin, taxifolin, and other polyphenolic components [96]; these are known compounds with antioxidant activity [97]. Several pharmacological actions of silybin were identified, including antioxidant and anti-inflammatory properties, antifibrotic effects, and the modulation of insulin resistance. Specifically, in chronic inflammation, it underlies liver fibrosis and the development of cirrhosis [98].

The common mechanism underlying the initiation and progression of liver inflammation is oxidative stress [99]. NF-κB is a crucial transcriptional regulator involved in the inflammatory response and plays an essential role in controlling the inflammatory signaling pathways within the liver [100]. Furthermore, NF-κB is activated in virtually all chronic liver diseases, including ALD, NAFLD, viral hepatitis, and biliary liver disease [101,102,103]. There is increasing evidence for a general inhibition of inflammatory mediators such as NF-κB and inflammatory metabolites (e.g., prostaglandin E2 (PGE 2) and leukotriene B4 (LTB 4)) by silymarin [104].

In isolated rat Kupffer cells, silymarin demonstrated a weak inhibition of PGE2 formation, but showed a strong ability to block the biosynthesis of LTB4 [105]. This selective inhibition of LTB 4 formation by Kupffer cells and possibly other cell types may explain silymarin’s anti-inflammatory potential.

In addition, it can reduce or normalize the liver function parameters, such as transaminase levels, and improve the ultrasound parameters of liver anatomy [106]. Therefore, silymarin (formulation derived from Eurosil 85^®^) was investigated as a therapeutic option for NAFLD and NASH (Table 1).

Therefore, silybin a has antioxidant and anti-inflammatory properties and may inhibit NF-κB and inflammatory metabolites, having potential to be a therapy for NAFLD and NASH, since it improves liver function and multiple ultrasound parameters used in liver ultrasonography. Silybin holds promise as a therapeutic agent for liver inflammation and fibrosis.

### 3.3. Breviscapine

Breviscapine is a crude mixture of several flavonoids from the traditional Chinese herb *Erigeron breviscapus*. It consists of more than 90% scutellarin and also contains baicalein, naringenin, scopoletin, kaempferol, apigenin, scutellarin, luteolin, caffeic acid, and protocatechuic acid [107]. It has multiple pharmacological activities, including anti-inflammatory, antioxidant, antiapoptotic, vasorelaxant, antiplatelet, anticoagulant, and myocardial protective activities [108,109]. Recent studies show that breviscapine protects against CCl_4_-induced liver injury by reducing proinflammatory cytokine secretion and oxidative stress [110]. In addition, scutellarin, the main component of breviscapine, was shown to regulate lipid metabolism and reduce oxidative stress in NAFLD [111].

Further studies show that in mice that are fed a high-fat diet (HFD), high-fat/high-cholesterol diet (HFHC), or methionine- and choline-deficient diet (MCD), breviscapine attenuates hepatic lipid accumulation, inflammation, and fibrosis by mediating effects via the direct inhibition of TGF-β-activated kinase 1 (TAK1) MAPKKK (mitogen-activated protein kinase kinase kinase).

TAK1, besides being an upstream protein kinase in the MAPK signaling pathway, also plays a role in activating NF-κB during the progression of NAFLD [112]. Because of the pivotal role of TAK1 in many physiological processes, such as inflammation, cell differentiation, and apoptosis, the inhibition of metabolic stress-induced TAK1 activation may provide a potent hepatoprotective effect [113]. Indeed, recent studies highlighted the important role of TAK1 in regulating hepatocyte lipid metabolism and reducing hepatocyte inflammation in NAFLD and NASH [114,115] (Table 1).

### 3.4. ALS-L1023

ALS-L1023 is an extract isolated from *Melissa officinalis*, known in natural medicine as an anti-angiogenic agent [116,117,118,119]. ALS-L1023 modulates the mRNA expression of angiogenic factors (VEGF-A and fibroblast growth factor 2 (FGF-2)), metalloproteinases (MMPs) MMP-2 and MMP-9, and their inhibitors (TIMP-1, TIMP-2, and thrombospondin (TSP-1)) [120,121]. It also reduces visceral adipose tissue (VAT) production in mice with HFD-induced NAFLD by suppressing lipid synthesis and steatosis, inflammatory cell infiltration, and collagen accumulation in the liver, thus supporting the idea that ALS-L1023 may have a great contribution to regulate the development and progression of NAFLD and consequent NASH [117,118]. In addition, this extract reduced inflammation in female ovariectomized mice with diet-induced NAFLD and rescued them from oxidative stress through Akt activation. For these reasons, ALS-L1023 is in a Phase IIa clinical trial involving patients with NAFLD. Recent studies are also evaluating whether ALS-L1023 could alleviate liver fibrosis, as antifibrotic effects are essential properties of NASH drugs. In this regard, a biochemical analysis showed that the ALS-L1023 mouse group had significantly decreased alanine transaminase and aspartate transaminase. In addition, the area of fibrosis was significantly reduced after the administration of ALS-L1023, and its antifibrotic effect was greater than that of the reference compound OCA (obeticholic acid) [122] (Table 1). ALS-L1023 has anti-angiogenic and anti-inflammatory properties, reduces VAT production and inflammation in NAFLD, and alleviates liver fibrosis, with a significative antifibrotic effect.

### 3.5. Curcumin

Curcumin, along with demethoxycurcumin, bisdemethoxycurcumin, and cyclocurcumin, is the major phenylpropanoid compound that is found in the roots of the perennial plant *Curcuma longa*. It is unclear whether all four analogs have the same activity. Although curcumin was found to be the most potent in most systems [123,124,125,126], bisdemethoxycurcumin and cyclocurcumin were found to have higher activity in some studies [127,128]. There is also evidence that the mixture of all three is more potent than just one [128]. Curcumin has shown anti-angiogenic and other beneficial properties in several experimental models of liver injury by inhibiting the NF-κB pathway [129]. It prevents aflatoxin-induced liver injury [130] and regresses cirrhosis [131].

In acute CCl_4_ intoxication, oxidative stress and the expression of pro-inflammatory cytokines, such as TNF-α, IL-1β, and IL-6, and NF-κB activation are associated with some increases in several markers of liver injury and the distortion of hepatic microscopic structure; pretreatment with curcumin prevented oxidative stress, NF-κB activation, and liver injury. Similarly, in biliary cirrhosis, curcumin showed anti-fibrogenic properties associated with the downregulation of the cytokine TGF-β [132] (Table 1).

NAFLD induced in methionine- and choline-deficient (MCD) mice significantly upregulates the hexosamine biosynthetic pathway and O-GlcNAc transferase via ER stress. This model was shown to be a useful experimental model for NASH. Curcumin treatment alleviates the severity of hepatic steatosis by blocking the NF-κB signaling pathway through the inhibition of O-GlcNAcylation and enhancing antioxidant systems, including SOD1 (superoxide dismutase 1), GPx (glutathione peroxidase), and CAT (catalase) [133] (Table 1).

### 3.6. Sulforaphane

Sulforaphane (SFN) is an organosulfur compound in the isothiocyanate group that is found in high concentrations in cruciferous vegetables such as broccoli and cauliflower [134]. It was reported to exert a variety of bioactive effects, including antioxidant, anti-inflammatory, cytotoxic, and cytoprotective effects [135]. In tumor angiogenesis, SFN inhibits NF-κB-regulated VEGF expression in human prostate cancer cells [136]; Peng liu et al. showed that SFN interferes with the proliferation, migration, and tube formation of endothelial cells, but it is also capable of inhibiting the pro-angiogenic effect of HepG2 cells both in vitro, ex vivo, and in vivo. SFN significantly inhibits the viability and migration of HUVECs (human umbilical vein endothelial cells), which is consistent with previous findings [137].

SFN was shown to inhibit STAT3 (signal transducer and activator of transcription 3), and this is consistent with the reduced expression of HIF-1α and VEGF in HepG2, suggesting that STAT3/HIF-1α/VEGF may be responsible for the anti-angiogenic effects of SFN. The latter also inhibits the TGF-β-induced epithelial–mesenchymal transition of HCC via the ROS-dependent pathway [138]. SFN-induced Nrf2 activation was indeed confirmed by the mRNA upregulation of redox genes such as HO-1 (heme oxygenase-1), MRP2 (multidrug resistance-associated protein 2), and NQO1 (NAD(P)H quinone dehydrogenase 1) in several cell lines [139,140] (Table 1). Essentially, SFN is a bioactive compound with a range of beneficial effects, including antioxidant, anti-inflammatory, cytotoxic, and cytoprotective effects, which has been shown to inhibit angiogenesis in human prostate and liver cancer cells by inhibiting NF-κB-regulated VEGF expression, and through inhibiting the STAT3/HIF-1α/VEGF pathway. SFN also induces Nrf2 activation, which may contribute to its antitumor effects.

### 3.7. Cordycepin

Cordycepin is an adenosine derivative, antimetabolite, and antibiotic from the fungus *Cordyceps militaris* [141]. Cordycepin has a broad spectrum of biological activities, including anti-inflammatory, antioxidant, antifibrotic, antiadipogenic, and antitumor activities [142,143]. Guo Z. et al. showed that it suppresses the migration and invasion of human liver cancer cells by downregulating CXCR4 [144]. A hallmark of NASH pathogenesis is the inflammatory response, which initially triggers the release of numerous types of proinflammatory cytokines. Cordycepin suppresses the production of proinflammatory cytokines in LPS-stimulated macrophages and exerts anti-inflammatory effects by suppressing NLRP3 activity, which is an important regulator of the inflammatory process in macrophages [145]. Furthermore, NLRP3 deficiency in HCC cells enhances the activity of natural killer cells to delay tumor development in the xenograft mouse model [146].

Tian Lan et al. also demonstrated in their study how cordycepin attenuates metabolic stress-induced NASH by preventing steatosis, inflammation, and fibrosis. Specifically, cordycepin inhibits the activation of the NF-κB signaling pathway and attenuates the secretion of proinflammatory cytokines in hepatocytes under metabolic stress. This is due to an increase in AMPK phosphorylation through an interaction with the α-subunit of AMPK. The latter acts in mice treated with NASH-inducing diets as a central regulator of fatty acid, cholesterol, and glucose homeostasis through the phosphorylation of enzymes that regulate metabolism, including ACCα (acetyl-CoA carboxylase), glycogen synthase, glucose transporter 4, and HMG-CoA (3-hydroxy-3-methyl-glutaryl-CoA) reductase [147] (Table 1). In conclusion, cordycepin, an adenosine derivative, was shown to exhibit various biological activities, including anti-inflammatory, antioxidant, antifibrotic, anti-adipogenic, and antitumor effects. Cordycepin also enhances natural killer cell activity to delay tumor development in xenograft mice models. These findings suggest that cordycepin may have potential therapeutic applications in the treatment of liver diseases, including NASH and liver cancer.

### 3.8. Methoxyeugenol

Methoxyeugenol is a compound found in Brazilian red propolis, which is produced by *Apis mellifera* bees. Propolis has a long history in folk medicine and istraditionally used to treat infections, gastric disorders, and promote wound healing [148,149]. Another source of methoxyeugenol is nutmeg (*Myristica fragrans Houtt.*), which is used as a spice, and its use in folk medicine is reported mainly for the treatment of gastrointestinal disorders [150]. However, studies support its use in the treatment of nonalcoholic hepatic steatosis [151], as it appears to promote lipid metabolism regulation and exert anti-inflammatory effects. Bruno de Souza Basso et al. showed that methoxyeugenol promotes HSC deactivation without evidence of cell death, suggesting a reduction in the proliferative rate [152]. In addition, the cytokine TGF-β, a growth factor that plays an important role in the development of liver fibrosis, particularly by activating quiescent HSCs, is reduced in the same cells treated with methoxyeugenol.

Lipid metabolism is regulated by nuclear receptors known as the PPAR family, particularly, PPARα and PPARγ. Research suggests that activating PPARγ can suppress macrophage activation and inflammatory pathways mediated by NF-κB [153,154]. In light of this, PPARγ activation may enhance the phenotypic modulation of activated HSCs by restoring lipid metabolism and inhibiting inflammatory signaling. In this regard, methoxyeugenol increases PPARɣ mRNA expression. In vivo studies on the use of methoxyeugenol, administered in the CCl_4_-induced liver fibrosis model, attenuate the inflammatory process and fibrosis, reducing intralobular inflammation by decreasing the gene expression of TNF-α, IL-6, and IL-8, as well as NF-κB protein expression [152] (Table 1). Overall, methoxyeugenol shows promising potential as a natural compound for the treatment of liver diseases.

### 3.9. Naringenin

Naringenin is a flavone that is found in various plants and is particularly abundant in citrus fruits [155]. Several studies show that it exerts multiple biological effects, including anti-inflammatory, antioxidant, and hypolipidemic effects, which are protective factors for NAFLD [156,157].

The study by Wang et al. showed that the mRNA expression levels of NF-κB, IL-1β, IL-18, and NLRP3 were improved in mice that were fed an MCD diet. In vivo experiments showed that naringenin significantly reduced the mRNA and protein levels of these factors. In addition, the hepatic triglyceride levels were not further reduced in the NLRP3−/− mice that were fed an MCD diet treated with naringenin. For further confirmation, primary hepatocytes from wild-type and NLRP3−/− mice were isolated, and NLRP3−/− cells were stimulated via LPS and oleic acid. It was found that naringenin is highly effective in preventing lipid deposition in WT primary hepatocytes, but much less effective in NLRP3−/− primary hepatocytes, demonstrating that this flavonoid acts precisely by reducing the levels of the NLRP3 inflammasome [158] (Table 1).

### 3.10. Ferulic Acid

Ferulic acid (FA) is a phenolic acid that is widely found in grains, vegetables, and plants such as *Angelica sinensis*. FA exhibits several biological activities, including antioxidant, anticancer, antidiabetic, and immune function-enhancing effects [159,160].

Recent studies show that FA exhibits hepatoprotective effects [161]. Indeed, it improves lipid metabolism and reduces liver inflammation in apolipoprotein E-deficient mice that are fed a HFD by upregulating AMPKα and downregulating lipogenic genes [162]. In addition, FA shows beneficial effects against oxidative stress and liver damage by activating the Nrf2/HO-1 and PPARγ pathways [163].

Jianzhi Wu et al. reported FA as an agent that is capable of preventing all histological changes of CCl_4_-induced liver fibrosis in mice by suppressing hepatic oxidative stress, inflammatory response, macrophage activation, and HSC activation through the phosphorylation of AMPK via direct binding and the consequent inhibition of PTP1B (protein tyrosine phosphatase 1B) [164]. Therefore, activated AMPK in hepatocytes not only suppresses apoptosis and NOX2 (NADPH oxidase 2)-derived ROS production, but also inhibits the production of endothelial pro-inflammatory cytokines [165]. FA is also able to prevent the nuclear translocation of NF-κB. These benefits make FA a good player in the treatment of liver fibrosis and its advanced complications (Table 1).

### 3.11. Betaine

Betaine is an essential biochemical modulator of the methionine/homocysteine cycle that is originally extracted from the juice of sugar beet (*Beta vulgaris*) [166] and is also found in various microorganisms, plants, and animals [167]. Aqueous extracts of betaine are used in traditional Eastern medicine to treat liver disease [168]. Recent studies show that betaine dampens down inflammation through the activation of NF-κB during aging [169]. Eui-Yeun Yi et al. found that betaine suppresses angiogenesis in the in vivo Matrigel plug assay, in addition to the in vitro inhibition in tube formation, migration, and invasion assays performed on HUVECs. Betaine also inhibits NF-κB and Akt activation [170].

VEGF and FGF-2 are known to be key stimuli for angiogenesis [171,172]. When investigating the involvement of betaine in the expression of key angiogenic factors, it was found that the mRNA expression of FGF-2 was significantly reduced. Betaine also shows a beneficial effect of downregulating the expression of MMP-2 and MMP-9, which are key regulators of extracellular matrix turnover, through the degradation of a variety of extracellular matrix proteins. Extracellular proteolytic activity is important in the process of endothelial cell migration and invasion, which are focal events in angiogenesis [173,174] (Table 1). Therefore, betaine suppresses inflammation and angiogenesis by inhibiting NF-κB and Akt activation, reducing the expression of key angiogenic factors, and regulating the extracellular matrix turnover.

### 3.12. Catechins

Catechins are a group of flavanols that are abundant in plant fruits, vegetables, and beverages. Grapes, apples, cocoa, and green tea are considered the major sources of catechins, which represent some promising candidates in the field of biomedicine [175,176,177,178]. Green tea is a widely consumed beverage globally and has been extensively researched for its potential health benefits. Studies focused on its positive effects in various diseases, including cancer, obesity, diabetes, and cardiovascular disease [179]. Many of the biological effects of green tea are mediated by its polyphenolic catechins, particularly (-)-epigallocatechin-3-gallate (EGCG), which accounts for 50–85% of total catechins. Several studies show how green tea in animals restores gene and protein levels of proinflammatory cytokines in galactosamine-induced hepatitis [180]. Similar observations were reported in CCl_4_-induced liver injury in rodents [181], where the mRNA expression levels of TNF-α, COX-2, iNOS, smooth muscle actin, TGF-1, procollagen-I, MMP-2, MMP-9, and TIMPs increased along with an increase in NF-κB activity. Treatment with green tea extract significantly reduced liver damage, oxidative stress, inflammatory response, and expression of all the analyzed pro-fibrogenic markers except for TIMP-2 and MMP-9. Other studies show that treatment with such extract inhibits the levels of IL-1, IL-6, and TNF-α [182]. Furthermore, vascular endothelial (VE) cadherin and Akt, which are known downstream proteins in the VEGFR-2-mediated cascade, are other target proteins by which EGCG inhibits angiogenesis [183]. It was also observed that pentameric procyanidins isolated from cocoa inhibit the gene expression of the tyrosine kinase ErbB2, thereby slowing down the in vitro angiogenesis of human aortic endothelial cells [184] (Table 1).

### 3.13. Puerarin

Puerarin, a natural compound extracted from *Pueraria lobata*, has antioxidant and anti-inflammatory effects [185]. Various studies show that puerarin may regulate leptin signaling through the Janus kinase 2 (JAK2)/STAT3 pathway, thereby ameliorating hepatic steatosis [186]. Jingxuan Zhou et al. showed that in a rat model of HFD-induced NAFLD, puerarin administration reduced the abdominal fat coefficient [187]. In addition, puerarin significantly reduces inflammation caused by lipid accumulation. This induces ROS overproduction and the consequent activation of the NF-κB pathway, as well as a variety of inflammatory factors, such as interleukins (IL-1β and IL-18) and TNF-α [188]. In addition, puerarin decreased the thiobarbituric acid reactive substances and protein carbonyl content in the livers of CCl_4_-treated mice by regulating the expression of phosphorylated JNK, phosphorylated c-Jun protein, and cholesterol 7a hydroxylase (CYP7A1) in the liver [189] (Table 1).

### 3.14. Resveratrol

Resveratrol (RSV) is a stilbene that is found in grape skins, blueberries, raspberries, mulberries, and peanuts. Additionally, red wine is known for its high concentration of the RSV that partly explains the relatively low incidence of cardiovascular disease despite the prevalence of HFD in populations using it [190]. Resveratrol has antioxidant activity in a wide range of liver diseases [190,191]. In particular, it reduces the ROS levels, enhances the activity of antioxidant enzymes (e.g., SOD, CAT), and promotes the synthesis of antioxidant molecules. RSV also influences the expression of genes involved in mitochondrial energy production through signaling pathways such as AMPK/SIRT1/Nrf2, ERK/p38 (extracellular signal-regulated kinase), and PTEN/Akt (phosphatase and tensin homologue/protein kinase B) [192]. By inhibiting the ubiquitination of Nrf2, it helps to maintain its crucial role in activating the transcription of various antioxidant genes, such as SOD and CAT, thereby strengthening the overall antioxidant defense system [192].

In NAFLD, RSV appears to have a significant role in regulating the accumulation of fibrosis through its impact on multiple crucial pathways. Indeed, RSV administration reduces portal pressure and HSC activation, and improves hepatic endothelial function in cirrhotic rats, with an overall beneficial effect on cirrhosis and portal hypertension [193,194]. Again, it is well known that RSV administration in hepatocytes causes the inhibition of mRNA expression of inflammatory mediators, including TNF-α, IL-1β, as well as TNF-α and IL-6 in cultured hepatocyte cells, and reduces the number of Kupffer CD68(+) cells recruited to the liver [195,196] (Table 1).

### 3.15. Fucoidan

Fucoidan, a sulfated polysaccharide containing substantial amounts of L-fucose and sulfate ester groups, is readily present in edible brown seaweeds, which are widely consumed in Asian countries due to their extensive health benefits [197,198]. An ex vivo angiogenesis assay showed that fucoidan caused a significant reduction in microvessel outgrowth and significantly reduced the expression of the angiogenesis factor VEGF-A [199]. Fucoidan was also evaluated for its activity in combination with sorafenib and bevacizumab in HCC. In vitro, on Huh-7 cells, fucoidan showed a potent synergistic effect with anti-angiogenic drugs and significantly reduced the HCC cell line viability in a dose-dependent manner via the inhibition of the pro-angiogenic PI3K/AKT/mTOR and KRAS/BRAF/MAPK pathways [200] (Table 1).

### 3.16. Carnosol and Carnosic Acid

Carnosol and carnosic acid are two major components of rosemary extracts that contribute to the chemopreventive, anti-inflammatory, antitumor, and antimetastatic activities of *Rosmarinus officinalis* [201,202]. Lopez-Jimenez et al. demonstrated for the first time that these diterpenes modulate different relevant steps of the angiogenic process by inhibiting cytokine-induced adhesion molecule expression, monocyte adhesion to endothelial cells through a mechanism related to NF-κB, and capillary tube formation via endothelial cells, in addition to inducing apoptosis in endothelial and tumor cells in vitro. These results suggest their potential use in the treatment of angiogenesis-related malignancies [203] (Table 1).

**Table 1:** Anti-angiogenic effects of natural compounds and their molecular mechanism.

Phytochemicals	Main Dietary Sources	Designed Study	Anti-Angiogenic Effects	Refs.
**Quercetin**	Apples, red grapes, citrus fruits, tomatoes, onions, and green leafy vegetables	Mice	HMGB1/TLR2/4-NF-κB signaling pathway downregulation; TGF-β1/Smads and PI3K/Akt crosstalk regulation	[90,91]
**Silybin**	*Silybum marianum*	Rats	NF-κB, PGE 2, and LTB 4 inhibition	[100,104]
**Breviscapine**	*Erigeron breviscapus*	Mice	TGF-β-activated kinase 1 (TAK1)/NF-κB signaling pathway inhibition	[112]
**ALS-L1023**	*Melissa officinalis*	Mice	mRNA modulation of VEGF-A, FGF-2, MMP-2, MMP-9, TIMP-1, TIMP-2, and TSP-1	[120,121]
**Curcumin**	*Curcuma longa*	Mice	NF-κB inhibition; TGF-β downregulation	[129,132]
**Sulforaphane**	Broccoli, cauliflower	Hep G2 and HUVEC cells	NF-κB/VEGF, STAT3/HIF-1α/VEGF signaling pathway inhibition; upregulation of HO-1, NQO1, and MRP2 genes	[136,138,139,140]
**Cordycepin**	*Cordyceps militaris*	HCC cells	CXCR4 downregulation; NLRP3 suppression; NF-κB inhibition	[144,145,146]
**Methoxyeugenol**	Brazilian red propolis	Mice	mRNA upregulation of PPARγ; downregulation of TNF-α, IL-6, and IL-8 genes	[152]
**Naringenin**	Citrus fruits	Mice	mRNA downregulation of NF-κB, IL-1β, IL-18, and NLRP3	[158]
**Ferulic acid**	Cereals, vegetables, and plants such as *Angelica sinensis*	Mice	AMPKα upregulation; lipogenic genes downregulation; Nrf2/HO-1 and PPARγ pathways activation; nuclear translocation block of NF-κB; PTP1B inhibition	[162,163,164,165]
**Betaine**	*Beta vulgaris*	HUVEC cells	NF-κB and Akt inhibition; FGF-2, MMP-2, and MMP downregulation	[170,173,174]
**Catechins**	Grapes, apples, and cocoa	Haec cells; mice	IL-1, IL-6, and TNF-α inhibition; ErbB2 gene downregulation	[182,184]
**Puerarin**	*Pueraria lobata*	Rats	(JAK2)/STAT3 signaling pathway regulation; JNK activity regulation	[186,189]
**Resveratrol**	Grape skins, blueberries, raspberries, mulberries, and peanuts	Primary hepatocyte; mice	Nrf2 ubiquitination inhibition; TNF-α, IL-1β, and IL-6 mRNA inhibition	[192,195,196]
**Fucoidan**	Edible brown seaweeds	HCC; Huh-7 cells	VEGF-A gene downregulation	[199,200]
**Carnosol and** **Carnosic acid**	*Rosmarinus* *officinalis*	BAECs and HUVEC cells	NF-κB inhibition	[203]

## 4. Conclusions and Perspectives

Angiogenesis plays an important role in the development of liver disease, contributing to the progression and remodeling of fibrosis, leading to capillarization of the sinusoids with the formation of intrahepatic shunts. As a result, vascular resistance increases, and hepatocyte perfusion decreases, leading to hypoxia.

Many anti-angiogenic drugs, mainly tyrosine kinase inhibitors and humanized monoclonal antibodies, are currently used, but they are often expensive or toxic, which limits their use in many cases. Therefore, it is useful to identify less toxic, inexpensive, novel, and effective anti-angiogenic compounds. In this regard, several natural plant products have shown prominent anti-angiogenic effects. However, the bioavailability of natural products is a critical issue since many of them have poor aqueous solubility and a low absorption rate.

On the positive side, a large number of natural anti-angiogenic substances are being discovered, and the study of their complex mechanisms has not yet been fully investigated.

Therefore, it is of great interest to study natural compounds and their derivatives to gain new insights into the biochemical mechanisms involved in signaling pathways in liver diseases. Accordingly, research on new natural products with anti-angiogenic effects seems very promising and may lead to the discovery of new drugs, which would be a significant achievement in this field. Furthermore, this sets the stage for the potential to identify new targets for more selective and effective synthetic anti-angiogenic agents.

## Data Availability

Not applicable.

## References

[1] Trefts E., Gannon M., Wasserman D.H. (2017). The liver. Curr. Biol..

[2] Racanelli V., Rehermann B. (2006). The liver as an immunological organ. Hepatology.

[3] Ko S., Russell J.O., Molina L.M., Monga S.P. (2020). Liver Progenitors and Adult Cell Plasticity in Hepatic Injury and Repair: Knowns and Unknowns. Annu. Rev. Pathol..

[4] Ikejima K., Kon K., Yamashina S. (2020). Nonalcoholic fatty liver disease and alcohol-related liver disease: From clinical aspects to pathophysiological insights. Clin. Mol. Hepatol..

[5] Friedman S.L., Neuschwander-Tetri B.A., Rinella M., Sanyal A.J. (2018). Mechanisms of NAFLD development and therapeutic strategies. Nat. Med..

[6] Gilgenkrantz H., de l’Hortet A.C. (2018). Understanding Liver Regeneration: From Mechanisms to Regenerative Medicine. Am. J. Pathol..

[7] Nassir F., Rector R.S., Hammoud G.M., Ibdah J.A. (2015). Pathogenesis and Prevention of Hepatic Steatosis. Gastroenterol. Hepatol..

[8] Forbes S.J., Newsome P.N. (2016). Liver regeneration—Mechanisms and models to clinical application. Nat. Rev. Gastroenterol. Hepatol..

[9] Seth D., Haber P.S., Syn W.K., Diehl A.M., Day C.P. (2011). Pathogenesis of alcohol-induced liver disease: Classical concepts and recent advances. J. Gastroenterol. Hepatol..

[10] Li S., Tan H.Y., Wang N., Zhang Z.J., Lao L., Wong C.W., Feng Y. (2015). The Role of Oxidative Stress and Antioxidants in Liver Diseases. Int. J. Mol. Sci..

[11] Ceni E., Mello T., Galli A. (2014). Pathogenesis of alcoholic liver disease: Role of oxidative metabolism. World J. Gastroenterol..

[12] Quagliariello V., Basilicata M.G., Pepe G., De Anseris R., Di Mauro A., Scognamiglio G., Palma G., Vestuto V., Buccolo S., Luciano A. (2022). Combination of Spirulina platensis, Ganoderma lucidum and Moringa oleifera Improves Cardiac Functions and Reduces Pro-Inflammatory Biomarkers in Preclinical Models of Short-Term Doxorubicin-Mediated Cardiotoxicity: New Frontiers in Cardioncology?. J. Cardiovasc. Dev. Dis..

[13] Cao P., Sun J., Sullivan M.A., Huang X., Wang H., Zhang Y., Wang N., Wang K. (2018). Angelica sinensis polysaccharide protects against acetaminophen-induced acute liver injury and cell death by suppressing oxidative stress and hepatic apoptosis in vivo and in vitro. Int. J. Biol. Macromol..

[14] Chen A. (2002). Acetaldehyde stimulates the activation of latent transforming growth factor-beta1 and induces expression of the type II receptor of the cytokine in rat cultured hepatic stellate cells. Biochem. J..

[15] Parola M., Robino G. (2001). Oxidative stress-related molecules and liver fibrosis. J. Hepatol..

[16] Kaphalia B.S., Cai P., Khan M.F., Okorodudu A.O., Ansari G.A. (2004). Fatty acid ethyl esters: Markers of alcohol abuse and alcoholism. Alcohol.

[17] Cederbaum A.I. (2013). Nrf2 and antioxidant defense against CYP2E1 toxicity. Sub-Cell. Biochem..

[18] Lin Q., Kang X., Li X., Wang T., Liu F., Jia J., Jin Z., Xue Y. (2019). NF-κB-mediated regulation of rat CYP2E1 by two independent signaling pathways. PLoS ONE.

[19] Magne L., Blanc E., Legrand B., Lucas D., Barouki R., Rouach H., Garlatti M. (2011). ATF4 and the integrated stress response are induced by ethanol and cytochrome P450 2E1 in human hepatocytes. J. Hepatol..

[20] Hwang S., Yun H., Moon S., Cho Y.E., Gao B. (2021). Role of Neutrophils in the Pathogenesis of Nonalcoholic Steatohepatitis. Front. Endocrinol..

[21] Ali H., Assiri M.A., Shearn C.T., Fritz K.S. (2019). Lipid peroxidation derived reactive aldehydes in alcoholic liver disease. Curr. Opin. Toxicol..

[22] Abu-Freha N., Cohen B., Gordon M., Weissmann S., Fich A., Munteanu D., Yardeni D., Etzion O. (2023). Comorbidities and Malignancy among NAFLD Patients Compared to the General Population, A Nation-Based Study. Biomedicines.

[23] Kosmalski M., Śliwińska A., Drzewoski J. (2023). Non-Alcoholic Fatty Liver Disease or Type 2 Diabetes Mellitus—The Chicken or the Egg Dilemma. Biomedicines.

[24] Cusi K. (2009). Role of insulin resistance and lipotoxicity in non-alcoholic steatohepatitis. Clin. Liver Dis..

[25] Grønbaek H., Thomsen K.L., Rungby J., Schmitz O., Vilstrup H. (2008). Role of nonalcoholic fatty liver disease in the development of insulin resistance and diabetes. Expert Rev. Gastroenterol. Hepatol..

[26] Day C.P., James O.F. (1998). Steatohepatitis: A tale of two “hits”?. Gastroenterology.

[27] Mohammad S., Thiemermann C. (2021). Role of Metabolic Endotoxemia in Systemic Inflammation and Potential Interventions. Front. Immunol..

[28] Luukkonen P.K., Zhou Y., Sädevirta S., Leivonen M., Arola J., Orešič M., Hyötyläinen T., Yki-Järvinen H. (2016). Hepatic ceramides dissociate steatosis and insulin resistance in patients with non-alcoholic fatty liver disease. J. Hepatol..

[29] Adolph T.E., Grander C., Grabherr F., Tilg H. (2017). Adipokines and Non-Alcoholic Fatty Liver Disease: Multiple Interactions. Int. J. Mol. Sci..

[30] Vestuto V., Di Sarno V., Musella S., Di Dona G., Moltedo O., Gomez-Monterrey I.M., Bertamino A., Ostacolo C., Campiglia P., Ciaglia T. (2023). New Frontiers on ER Stress Modulation: Are TRP Channels the Leading Actors?. Int. J. Mol. Sci..

[31] Fabbrini E., Magkos F., Mohammed B.S., Pietka T., Abumrad N.A., Patterson B.W., Okunade A., Klein S. (2009). Intrahepatic fat, not visceral fat, is linked with metabolic complications of obesity. Proc. Natl. Acad. Sci. USA.

[32] Baffy G. (2009). Kupffer cells in non-alcoholic fatty liver disease: The emerging view. J. Hepatol..

[33] Grimaldi M., Palisi A., Marino C., Montoro P., Capasso A., Novi S., Tecce M.F., D’Ursi A.M. (2020). NMR-based metabolomic profile of hypercholesterolemic human sera: Relationship with in vitro gene expression?. PLoS ONE.

[34] Asif S., Kim R.Y., Fatica T., Sim J., Zhao X., Oh Y., Denoncourt A., Cheung A.C., Downey M., Mulvihill E.E. (2022). Hmgcs2-mediated ketogenesis modulates high-fat diet-induced hepatosteatosis. Mol. Metab..

[35] Czaja M.J. (2010). JNK regulation of hepatic manifestations of the metabolic syndrome. Trends Endocrinol. Metab..

[36] Najjar S.M., Abdolahipour R., Ghadieh H.E., Jahromi M.S., Najjar J.A., Abuamreh B.A.M., Zaidi S., Kumarasamy S., Muturi H.T. (2022). Regulation of Insulin Clearance by Non-Esterified Fatty Acids. Biomedicines.

[37] Polyzos S.A., Kountouras J., Zavos C. (2009). Nonalcoholic fatty liver disease: The pathogenetic roles of insulin resistance and adipocytokines. Curr. Mol. Med..

[38] Berger J., Moller D.E. (2002). The mechanisms of action of PPARs. Annu. Rev. Med..

[39] Montagner A., Polizzi A., Fouché E., Ducheix S., Lippi Y., Lasserre F., Barquissau V., Régnier M., Lukowicz C., Benhamed F. (2016). Liver PPARα is crucial for whole-body fatty acid homeostasis and is protective against NAFLD. Gut.

[40] Duan Y., Pan X., Luo J., Xiao X., Li J., Bestman P.L., Luo M. (2022). Association of Inflammatory Cytokines with Non-Alcoholic Fatty Liver Disease. Front. Immunol..

[41] Khan R.S., Bril F., Cusi K., Newsome P.N. (2019). Modulation of Insulin Resistance in Nonalcoholic Fatty Liver Disease. Hepatology.

[42] Syn W.K., Choi S.S., Diehl A.M. (2009). Apoptosis and cytokines in non-alcoholic steatohepatitis. Clin. Liver Dis..

[43] Auguet T., Bertran L., Binetti J., Aguilar C., Martínez S., Sabench F., Lopez-Dupla J.M., Porras J.A., Riesco D., Del Castillo D. (2020). Relationship between IL-8 Circulating Levels and TLR2 Hepatic Expression in Women with Morbid Obesity and Nonalcoholic Steatohepatitis. Int. J. Mol. Sci..

[44] Henao-Mejia J., Elinav E., Jin C., Hao L., Mehal W.Z., Strowig T., Thaiss C.A., Kau A.L., Eisenbarth S.C., Jurczak M.J. (2012). Inflammasome-mediated dysbiosis regulates progression of NAFLD and obesity. Nature.

[45] Baker R.G., Hayden M.S., Ghosh S. (2011). NF-κB, inflammation, and metabolic disease. Cell Metab..

[46] Oates J.R., McKell M.C., Moreno-Fernandez M.E., Damen M.S.M.A., Deepe G.S., Qualls J.E., Divanovic S. (2019). Macrophage Function in the Pathogenesis of Non-alcoholic Fatty Liver Disease: The Mac Attack. Front. Immunol..

[47] Buzzetti E., Pinzani M., Tsochatzis E.A. (2016). The multiple-hit pathogenesis of non-alcoholic fatty liver disease (NAFLD). Metab. Clin. Exp..

[48] Seki E., Schwabe R.F. (2015). Hepatic inflammation and fibrosis: Functional links and key pathways. Hepatology.

[49] Torres S., Segalés P., García-Ruiz C., Fernández-Checa J.C. (2022). Mitochondria and the NLRP3 Inflammasome in Alcoholic and Nonalcoholic Steatohepatitis. Cells.

[50] Seitz H.K., Bataller R., Cortez-Pinto H., Gao B., Gual A., Lackner C., Mathurin P., Mueller S., Szabo G., Tsukamoto H. (2018). Alcoholic liver disease. Nat. Rev. Dis. Prim..

[51] Llovet J.M., Kelley R.K., Villanueva A., Singal A.G., Pikarsky E., Roayaie S., Lencioni R., Koike K., Zucman-Rossi J., Finn R.S. (2021). Hepatocellular carcinoma. Nat. Rev. Dis. Prim..

[52] Yang Y.M., Cho Y.E., Hwang S. (2022). Crosstalk between Oxidative Stress and Inflammatory Liver Injury in the Pathogenesis of Alcoholic Liver Disease. Int. J. Mol. Sci..

[53] Dhar D., Baglieri J., Kisseleva T., Brenner D.A. (2020). Mechanisms of liver fibrosis and its role in liver cancer. Exp. Biol. Med..

[54] Pappachan J.M., Babu S., Krishnan B., Ravindran N.C. (2017). Non-alcoholic Fatty Liver Disease: A Clinical Update. J. Clin. Transl. Hepatol..

[55] Ratziu V., Bellentani S., Cortez-Pinto H., Day C., Marchesini G. (2010). A position statement on NAFLD/NASH based on the EASL 2009 special conference. J. Hepatol..

[56] Shan Z., Ju C. (2020). Hepatic Macrophages in Liver Injury. Front. Immunol..

[57] Wree A., McGeough M.D., Peña C.A., Schlattjan M., Li H., Inzaugarat M.E., Messer K., Canbay A., Hoffman H.M., Feldstein A.E. (2014). NLRP3 inflammasome activation is required for fibrosis development in NAFLD. J. Mol. Med..

[58] Papa S., Bubici C., Zazzeroni F., Franzoso G. (2009). Mechanisms of liver disease: Cross-talk between the NF-κB and JNK pathways. Biol. Chem..

[59] Lee J.S., Zheng Z., Mendez R., Ha S.W., Xie Y., Zhang K. (2012). Pharmacologic ER stress induces non-alcoholic steatohepatitis in an animal model. Toxicol. Lett..

[60] Brunt E.M., Tiniakos D.G. (2002). Pathology of steatohepatitis. Best Pract. Research. Clin. Gastroenterol..

[61] Tiniakos D.G., Vos M.B., Brunt E.M. (2010). Nonalcoholic fatty liver disease: Pathology and pathogenesis. Annu. Rev. Pathol..

[62] Wong V.W., Wong G.L., Choi P.C., Chan A.W., Li M.K., Chan H.Y., Chim A.M., Yu J., Sung J.J., Chan H.L. (2010). Disease progression of non-alcoholic fatty liver disease: A prospective study with paired liver biopsies at 3 years. Gut.

[63] Loomba R., Lawitz E., Mantry P.S., Jayakumar S., Caldwell S.H., Arnold H., Diehl A.M., Djedjos C.S., Han L., Myers R.P. (2018). GS-US-384-1497 Investigators. The ASK1 inhibitor selonsertib in patients with nonalcoholic steatohepatitis: A randomized, phase 2 trial. Hepatology.

[64] Bianco C., Casirati E., Malvestiti F., Valenti L. (2021). Genetic predisposition similarities between NASH and ASH: Identification of new therapeutic targets. JHEP Rep. Innov. Hepatol..

[65] Neuschwander-Tetri B.A. (2020). Therapeutic Landscape for NAFLD in 2020. Gastroenterology.

[66] Philip Esteban J., Dinani A. (2020). Lifestyle Interventions Beyond Diet and Exercise for Patients with Nonalcoholic Fatty Liver Disease. Gastroenterol. Hepatol..

[67] Norata G.D., Pellegatta F., Catapano A.L. (2003). “Peroxisome proliferator activated receptors” e patologie cardiovascolari [Peroxisome proliferator activated receptors and cardiovascular disorders]. Ital. Heart J. Suppl. Off. J. Ital. Fed. Cardiol..

[68] Loria P., Adinolfi L.E., Bellentani S., Bugianesi E., Grieco A., Fargion S., Gasbarrini A., Loguercio C., Lonardo A., Marchesini G. (2010). NAFLD Expert Committee of the Associazione Italiana per lo studio del Fegato Practice guidelines for the diagnosis and management of nonalcoholic fatty liver disease. A decalogue from the Italian Association for the Study of the Liver (AISF) Expert Committee. Dig. Liver Dis. Off. J. Ital. Soc. Gastroenterol. Ital. Assoc. Study Liver.

[69] Seaman S., Stevens J., Yang M.Y., Logsdon D., Graff-Cherry C., St Croix B. (2007). Genes that distinguish physiological and pathological angiogenesis. Cancer Cell.

[70] Bocca C., Novo E., Miglietta A., Parola M. (2015). Angiogenesis and Fibrogenesis in Chronic Liver Diseases. Cell. Mol. Gastroenterol. Hepatol..

[71] Fernández M., Semela D., Bruix J., Colle I., Pinzani M., Bosch J. (2009). Angiogenesis in liver disease. J. Hepatol..

[72] Camenisch G., Pisabarro M.T., Sherman D., Kowalski J., Nagel M., Hass P., Xie M.H., Gurney A., Bodary S., Liang X.H. (2002). ANGPTL3 stimulates endothelial cell adhesion and migration via integrin alpha vbeta 3 and induces blood vessel formation in vivo. J. Biol. Chem..

[73] Valfrè di Bonzo L., Novo E., Cannito S., Busletta C., Paternostro C., Povero D., Parola M. (2009). Angiogenesis and liver fibrogenesis. Histol. Histopathol..

[74] Baumeister S.E., Völzke H., Marschall P., John U., Schmidt C.O., Flessa S., Alte D. (2008). Impact of fatty liver disease on health care utilization and costs in a general population: A 5-year observation. Gastroenterology.

[75] Nath B., Szabo G. (2012). Hypoxia and hypoxia inducible factors: Diverse roles in liver diseases. Hepatology.

[76] Kukla M. (2013). Angiogenesis: A phenomenon which aggravates chronic liver disease progression. Hepatol. Int..

[77] Parola M., Marra F., Pinzani M. (2008). Myofibroblast-like cells and liver fibrogenesis: Emerging concepts in a rapidly moving scenario. Mol. Asp. Med..

[78] Lei L., Ei Mourabit H., Housset C., Cadoret A., Lemoinne S. (2021). Role of Angiogenesis in the Pathogenesis of NAFLD. J. Clin. Med..

[79] Coulon S., Heindryckx F., Geerts A., Van Steenkiste C., Colle I., Van Vlierberghe H. (2011). Angiogenesis in chronic liver disease and its complications. Liver Int. Off. J. Int. Assoc. Study Liver.

[80] Dooley S., Ten Dijke P. (2012). TGF-β in progression of liver disease. Cell Tissue Res..

[81] Zhu B., Lin N., Zhang M., Zhu Y., Cheng H., Chen S., Ling Y., Pan W., Xu R. (2015). Activated hepatic stellate cells promote angiogenesis via interleukin-8 in hepatocellular carcinoma. J. Transl. Med..

[82] Copple B.L., Bai S., Burgoon L.D., Moon J.O. (2011). Hypoxia-inducible factor-1α regulates the expression of genes in hypoxic hepatic stellate cells important for collagen deposition and angiogenesis. Liver Int. Off. J. Int. Assoc. Study Liver.

[83] Weiskirchen R., Meurer S.K., Liedtke C., Huber M. (2019). Mast Cells in Liver Fibrogenesis. Cells.

[84] Raevens S., Coulon S., Van Steenkiste C., Colman R., Verhelst X., Van Vlierberghe H., Geerts A., Perkmann T., Horvatits T., Fuhrmann V. (2015). Role of angiogenic factors/cell adhesion markers in serum of cirrhotic patients with hepatopulmonary syndrome. Liver Int. Off. J. Int. Assoc. Study Liver.

[85] Bedogni G., Miglioli L., Masutti F., Castiglione A., Crocè L.S., Tiribelli C., Bellentani S. (2007). Incidence and natural course of fatty liver in the general population: The Dionysos study. Hepatology.

[86] Lee C.K., Lee M.E., Lee W.S., Kim J.M., Park K.H., Kim T.S., Lee K.Y., Ahn J.B., Chung H.C., Rha S.Y. (2015). Dovitinib (TKI258), a Multi-Target Angiokinase Inhibitor, Is Effective Regardless of KRAS or BRAF Mutation Status in Colorectal Cancer. Am. J. Cancer Res..

[87] Lopes-Coelho F., Martins F., Pereira S.A., Serpa J. (2021). Anti-Angiogenic Therapy: Current Challenges and Future Perspectives. Int. J. Mol. Sci..

[88] Zhu X.D., Tang Z.Y., Sun H.C. (2020). Targeting angiogenesis for liver cancer: Past, present, and future. Genes Dis..

[89] Russo M., Spagnuolo C., Tedesco I., Bilotto S., Russo G.L. (2012). The flavonoid quercetin in disease prevention and therapy: Facts and fancies. Biochem. Pharmacol..

[90] Hisaka T., Sakai H., Sato T., Goto Y., Nomura Y., Fukutomi S., Fujita F., Mizobe T., Nakashima O., Tanigawa M. (2020). Quercetin Suppresses Proliferation of Liver Cancer Cell Lines In Vitro. Anticancer. Res..

[91] Li X., Liu H.C., Yao Q.Y., Xu B.L., Zhang S.C., Tu C.T. (2016). Quercetin Protects Mice from ConA-Induced Hepatitis by Inhibiting HMGB1-TLR Expression and Down-Regulating the Nuclear Factor Kappa B Pathway. Inflammation.

[92] Wu L., Zhang Q., Mo W., Feng J., Li S., Li J., Liu T., Xu S., Wang W., Lu X. (2017). Quercetin prevents hepatic fibrosis by inhibiting hepatic stellate cell activation and reducing autophagy via the TGF-β1/Smads and PI3K/Akt pathways. Sci. Rep..

[93] Sica A., Invernizzi P., Mantovani A. (2014). Macrophage plasticity and polarization in liver homeostasis and pathology. Hepatology.

[94] Tacke F., Zimmermann H.W. (2014). Macrophage heterogeneity in liver injury and fibrosis. J. Hepatol..

[95] Pradere J.P., Kluwe J., De Minicis S., Jiao J.J., Gwak G.Y., Dapito D.H., Jang M.K., Guenther N.D., Mederacke I., Friedman R. (2013). Hepatic macrophages but not dendritic cells contribute to liver fibrosis by promoting the survival of activated hepatic stellate cells in mice. Hepatology.

[96] Kren V., Walterová D. (2005). Silybin and silymarin—New effects and applications. Biomed. Pap. Med. Fac. Univ. Palacky Olomouc Czechoslov..

[97] Javed S., Kohli K., Ali M. (2011). Reassessing bioavailability of silymarin. Altern. Med. Rev..

[98] Surai P.F. (2015). Silymarin as a Natural Antioxidant: An Overview of the Current Evidence and Perspectives. Antioxidants.

[99] Younossi Z.M., Koenig A.B., Abdelatif D., Fazel Y., Henry L., Wymer M. (2016). Global epidemiology of nonalcoholic fatty liver disease-Meta-analytic assessment of prevalence, incidence, and outcomes. Hepatology.

[100] Hahn G., Lehmann H.D., Kürten M., Uebel H., Vogel G. (1968). Zur Pharmakologie und Toxikologie von Silymarin, des antihepatotoxischen Wirkprinzipes aus *Silybum marianum* (L.) Gaertn [On the pharmacology and toxicology of silymarin, an antihepatotoxic active principle from *Silybum marianum* (L.) Gaertn]. Arzneim. Forsch..

[101] Kondylis V., Kumari S., Vlantis K., Pasparakis M. (2017). The interplay of IKK, NF-κB and RIPK1 signaling in the regulation of cell death, tissue homeostasis and inflammation. Immunol. Rev..

[102] Gobejishvili L., Barve S., Joshi-Barve S., Uriarte S., Gobejishvili L., Barve S., Joshi-Barve S., Uriarte S., Song Z., McClain C. (2006). Chronic ethanol-mediated decrease in cAMP primes macrophages to enhanced LPS-inducible NF-κB activity and TNF expression: Relevance to alcoholic liver disease. Am. J. Physiology. Gastrointest. Liver Physiol..

[103] Guan Y.S., He Q., Wang M.Q., Li P. (2008). Nuclear factor kappa B and hepatitis viruses. Expert Opin. Ther. Targets.

[104] Muriel P. (2009). NF-κB in liver diseases: A target for drug therapy. J. Appl. Toxicol..

[105] Manna S.K., Mukhopadhyay A., Van N.T., Aggarwal B.B. (1999). Silymarin suppresses TNF-induced activation of NF-κB, c-Jun N-terminal kinase, and apoptosis. J. Immunol..

[106] Dehmlow C., Erhard J., de Groot H. (1996). Inhibition of Kupffer cell functions as an explanation for the hepatoprotective properties of silibinin. Hepatology.

[107] Trubitsyna I.E., Chikunova B.Z., Tkachenko E.V., Tsaregorodtseva T.M., Vinokurova L.V., Varvanina G.G. (2008). Pathophysiology of hormonal, immune, metabolic changes in acute and chronic pancreatitis. Experimental and clinical studies. Eksp. Klin. Gastroenterol..

[108] Pengyue Z., Tao G., Hongyun H., Liqiang Y., Yihao D. (2017). Breviscapine confers a neuroprotective efficacy against transient focal cerebral ischemia by attenuating neuronal and astrocytic autophagy in the penumbra. Biomed. Pharmacother..

[109] Lin L.L., Liu A.J., Liu J.G., Yu X.H., Qin L.P., Su D.F. (2007). Protective effects of scutellarin and breviscapine on brain and heart ischemia in rats. J. Cardiovasc. Pharmacol..

[110] Wang L., Ma Q. (2018). Clinical benefits and pharmacology of scutellarin: A comprehensive review. Pharmacol. Ther..

[111] Liu Y., Wen P.H., Zhang X.X., Dai Y., He Q. (2018). Breviscapine ameliorates CCl4-induced liver injury in mice through inhibiting inflammatory apoptotic response and ROS generation. Int. J. Mol. Med..

[112] Fan H., Ma X., Lin P., Kang Q., Zhao Z., Wang L., Sun D., Cheng J., Li Y. (2017). Scutellarin Prevents Nonalcoholic Fatty Liver Disease (NAFLD) and Hyperlipidemia via PI3K/AKT-Dependent Activation of Nuclear Factor (Erythroid-Derived 2)-Like 2 (Nrf2) in Rats. Med. Sci. Monit. Int. Med. J. Exp. Clin. Res..

[113] Sun H., Wang X., Chen J., Song K., Gusdon A.M., Li L., Bu L., Qu S. (2016). Melatonin improves non-alcoholic fatty liver disease via MAPK-JNK/P38 signaling in high-fat-diet-induced obese mice. Lipids Health Dis..

[114] Wu Y.K., Hu L.F., Lou D.S., Wang B.C., Tan J. (2020). Targeting DUSP16/TAK1 signaling alleviates hepatic dyslipidemia and inflammation in high fat diet (HFD)-challenged mice through suppressing JNK MAPK. Biochem. Biophys. Res. Commun..

[115] Wang J., Ma J., Nie H., Zhang X.J., Zhang P., She Z.G., Li H., Ji Y.X., Cai J. (2021). Hepatic Regulator of G Protein Signaling 5 Ameliorates Nonalcoholic Fatty Liver Disease by Suppressing Transforming Growth Factor Beta-Activated Kinase 1-c-Jun-N-Terminal Kinase/p38 Signaling. Hepatology.

[116] Shen X., Guo H., Xu J., Wang J. (2019). Inhibition of lncRNA HULC improves hepatic fibrosis and hepatocyte apoptosis by inhibiting the MAPK signaling pathway in rats with nonalcoholic fatty liver disease. J. Cell. Physiol..

[117] Park B.Y., Lee H., Woo S., Yoon M., Kim J., Hong Y., Lee H.S., Park E.K., Hahm J.C., Kim J.W. (2015). Reduction of Adipose Tissue Mass by the Angiogenesis Inhibitor ALS-L1023 from Melissa officinalis. PLoS ONE.

[118] Kim J., Lee H., Lim J., Oh J., Shin S.S., Yoon M. (2017). The Angiogenesis Inhibitor ALS-L1023 from Lemon-Balm Leaves Attenuates High-Fat Diet-Induced Nonalcoholic Fatty Liver Disease through Regulating the Visceral Adipose-Tissue Function. Int. J. Mol. Sci..

[119] Lee E.K., Kim Y.J., Kim J.Y., Song H.B., Yu H.G. (2014). Melissa officinalis extract inhibits laser-induced choroidal neovascularization in a rat model. PLoS ONE.

[120] Sipos S., Moacă E.-A., Pavel I.Z., Avram Ş., Crețu O.M., Coricovac D., Racoviceanu R.-M., Ghiulai R., Pană R.D., Şoica C.M. (2021). *Melissa officinalis* L. Aqueous Extract Exerts Antioxidant and Antiangiogenic Effects and Improves Physiological Skin Parameters. Molecules.

[121] Woo S., Yoon M., Kim J., Hong Y., Kim M.Y., Shin S.S., Yoon M. (2016). The anti-angiogenic herbal extract from *Melissa officinalis* inhibits adipogenesis in 3T3-L1 adipocytes and suppresses adipocyte hypertrophy in high fat diet-induced obese C57BL/6J mice. J. Ethnopharmacol..

[122] Kim J., Lee H., Lim J., Lee H., Yoon S., Shin S.S., Yoon M. (2017). The lemon balm extract ALS-L1023 inhibits obesity and nonalcoholic fatty liver disease in female ovariectomized mice. Food Chem. Toxicol..

[123] Lee E.J., Kim Y., Kim J.E., Yoon E.L., Lee S.R., Jun D.W. (2023). ALS-L1023 from *Melissa officinalis* Alleviates Liver Fibrosis in a Non-Alcoholic Fatty Liver Disease Model. Life.

[124] Howells L.M., Iwuji C.O.O., Irving G.R.B., Barber S., Walter H., Sidat Z., Griffin-Teall N., Singh R., Foreman N., Patel S.R. (2019). Curcumin Combined with FOLFOX Chemotherapy Is Safe and Tolerable in Patients with Metastatic Colorectal Cancer in a Randomized Phase IIa Trial. J. Nutr..

[125] Marino P., Pepe G., Basilicata M.G., Vestuto V., Marzocco S., Autore G., Procino A., Gomez-Monterrey I.M., Manfra M., Campiglia P. (2023). Potential Role of Natural Antioxidant Products in Oncological Diseases. Antioxidants.

[126] Longobardi C., Damiano S., Andretta E., Prisco F., Russo V., Pagnini F., Florio S., Ciarcia R. (2021). Curcumin Modulates Nitrosative Stress, Inflammation, and DNA Damage and Protects against Ochratoxin A-Induced Hepatotoxicity and Nephrotoxicity in Rats. Antioxidants.

[127] Damiano S., Longobardi C., Andretta E., Prisco F., Piegari G., Squillacioti C., Montagnaro S., Pagnini F., Badino P., Florio S. (2021). Antioxidative Effects of Curcumin on the Hepatotoxicity Induced by Ochratoxin A in Rats. Antioxidants.

[128] Randino R., Grimaldi M., Persico M., De Santis A., Cini E., Cabri W., Riva A., D’Errico G., Fattorusso C., D’Ursi A.M. (2016). Investigating the Neuroprotective Effects of Turmeric Extract: Structural Interactions of β-Amyloid Peptide with Single Curcuminoids. Sci. Rep..

[129] Vera-Ramirez L., Pérez-Lopez P., Varela-Lopez A., Ramirez-Tortosa M., Battino M., Quiles J.L. (2013). Curcumin and liver disease. BioFactors.

[130] Bhandarkar S.S., Arbiser J.L. (2007). Curcumin as an inhibitor of angiogenesis. Adv. Exp. Med. Biol..

[131] Damiano S., Jarriyawattanachaikul W., Girolami F., Longobardi C., Nebbia C., Andretta E., Lauritano C., Dabbou S., Avantaggiato G., Schiavone A. (2022). Curcumin Supplementation Protects Broiler Chickens against the Renal Oxidative Stress Induced by the Dietary Exposure to Low Levels of Aflatoxin B1. Front. Vet. Sci..

[132] Farzaei M.H., Zobeiri M., Parvizi F., El-Senduny F.F., Marmouzi I., Coy-Barrera E., Naseri R., Nabavi S.M., Rahimi R., Abdollahi M. (2018). Curcumin in Liver Diseases: A Systematic Review of the Cellular Mechanisms of Oxidative Stress and Clinical Perspective. Nutrients..

[133] Zhao Y., Ma X., Wang J., He X., Hu Y., Zhang P., Wang R., Li R., Gong M., Luo S. (2014). Curcumin protects against CCl4-induced liver fibrosis in rats by inhibiting HIF-1α through an ERK-dependent pathway. Molecules.

[134] Lee D.E., Lee S.J., Kim S.J., Lee H.S., Kwon O.S. (2019). Curcumin Ameliorates Nonalcoholic Fatty Liver Disease through Inhibition of O-GlcNAcylation. Nutrients.

[135] Nakagawa K., Umeda T., Higuchi O., Tsuzuki T., Suzuki T., Miyazawa T. (2006). Evaporative light-scattering analysis of sulforaphane in broccoli samples: Quality of broccoli products regarding sulforaphane contents. J. Agric. Food Chem..

[136] Kaiser A.E., Baniasadi M., Giansiracusa D., Giansiracusa M., Garcia M., Fryda Z., Wong T.L., Bishayee A. (2021). Sulforaphane: A Broccoli Bioactive Phytocompound with Cancer Preventive Potential. Cancers.

[137] Xu C., Shen G., Chen C., Gélinas C., Kong A.N. (2005). Suppression of NF-κB and NF-κB-regulated gene expression by sulforaphane and PEITC through IkappaBalpha, IKK pathway in human prostate cancer PC-3 cells. Oncogene.

[138] Davis R., Singh K.P., Kurzrock R., Shankar S. (2009). Sulforaphane inhibits angiogenesis through activation of FOXO transcription factors. Oncol. Rep..

[139] Hahm E.R., Singh S.V. (2010). Sulforaphane inhibits constitutive and interleukin-6-induced activation of signal transducer and activator of transcription 3 in prostate cancer cells. Cancer Prev. Res..

[140] Noh J.R., Kim Y.H., Hwang J.H., Choi D.H., Kim K.S., Oh W.K., Lee C.H. (2015). Sulforaphane protects against acetaminophen-induced hepatotoxicity. Food Chem. Toxicol..

[141] Sato S., Moriya K., Furukawa M., Saikawa S., Namisaki T., Kitade M., Kawaratani H., Kaji K., Takaya H., Shimozato N. (2018). Sulforaphane Inhibits Liver Cancer Cell Growth and Angiogenesis. Ann. Behav. Sci..

[142] Tuli H.S., Sharma A.K., Sandhu S.S., Kashyap D. (2013). Cordycepin: A bioactive metabolite with therapeutic potential. Life Sci..

[143] Cao T., Xu R., Xu Y., Liu Y., Qi D., Wan Q. (2019). The protective effect of Cordycepin on diabetic nephropathy through autophagy induction in vivo and in vitro. Int. Urol. Nephrol..

[144] Yoon S.Y., Park S.J., Park Y.J. (2018). The Anticancer Properties of Cordycepin and Their Underlying Mechanisms. Int. J. Mol. Sci..

[145] Guo Z., Chen W., Dai G., Huang Y. (2020). Cordycepin suppresses the migration and invasion of human liver cancer cells by downregulating the expression of CXCR4. Int. J. Mol. Med..

[146] Yang J., Li Y.Z., Hylemon P.B., Zhang L.Y., Zhou H.P. (2017). Cordycepin inhibits LPS-induced inflammatory responses by modulating NOD-Like Receptor Protein 3 inflammasome activation. Biomed. Pharmacother. Biomed. Pharmacother..

[147] Lee H.H., Kim D., Jung J., Kang H., Cho H. (2021). NLRP3 Deficiency in Hepatocellular Carcinoma Enhances Surveillance of NK-92 through a Modulation of MICA/B. Int. J. Mol. Sci..

[148] Lan T., Yu Y., Zhang J., Li H., Weng Q., Jiang S., Tian S., Xu T., Hu S., Yang G. (2021). Cordycepin Ameliorates Nonalcoholic Steatohepatitis by Activation of the AMP-Activated Protein Kinase Signaling Pathway. Hepatology.

[149] Costa B.P., Nassr M.T., Diz F.M., Fernandes K.H.A., Antunes G.L., Grun L.K., Barbé-Tuana F.M., Nunes F.B., Branchini G., de Oliveira J.R. (2021). Methoxyeugenol regulates the p53/p21 pathway and suppresses human endometrial cancer cell proliferation. J. Ethnopharmacol..

[150] Antunes G.L., Matzenbacher L.S., Costa B.P., de Sousa Basso B., Levorse V.G.S., Antunes K.H., Costa-Ferro Z.S.M., de Oliveira J.R. (2022). Methoxyeugenol Protects Against Lung Inflammation and Suppresses Neutrophil Extracellular Trap Formation in an LPS-Induced Acute Lung Injury Model. Inflammation.

[151] Hoda S., Vermani M., Joshi R.K., Shankar J., Vijayaraghavan P. (2020). Anti-melanogenic activity of Myristica fragrans extract against *Aspergillus fumigatus* using phenotypic based screening. BMC Complement. Med. Ther..

[152] Zhao W., Song F., Hu D., Chen H., Zhai Q., Lu W., Zhao J., Zhang H., Chen W., Gu Z. (2020). The Protective Effect of *Myristica fragrans* Houtt. Extracts Against Obesity and Inflammation by Regulating Free Fatty Acids Metabolism in Nonalcoholic Fatty Liver Disease. Nutrients..

[153] de Souza Basso B., Haute G.V., Ortega-Ribera M., Luft C., Antunes G.L., Bastos M.S., Carlessi L.P., Levorse V.G., Cassel E., Donadio M.V.F. (2021). Methoxyeugenol deactivates hepatic stellate cells and attenuates liver fibrosis and inflammation through a PPAR-ɣ and NF-κB mechanism. J. Ethnopharmacol..

[154] Mirza A.Z., Althagafi I.I., Shamshad H. (2019). Role of PPAR receptor in different diseases and their ligands: Physiological importance and clinical implications. Eur. J. Med. Chem..

[155] Kim J.H., Song J., Park K.W. (2015). The multifaceted factor peroxisome proliferator-activated receptor γ (PPARγ) in metabolism, immunity, and cancer. Arch. Pharmacal Res..

[156] Orhan I.E., Nabavi S.F., Daglia M., Tenore G.C., Mansouri K., Nabavi S.M. (2015). Naringenin and atherosclerosis: A review of literature. Curr. Pharm. Biotechnol..

[157] Wali A.F., Rashid S., Rashid S.M., Ansari M.A., Khan M.R., Haq N., Alhareth D.Y., Ahmad A., Rehman M.U. (2020). Naringenin Regulates Doxorubicin-Induced Liver Dysfunction: Impact on Oxidative Stress and Inflammation. Plants.

[158] Zhao M., Li C., Shen F., Wang M., Jia N., Wang C. (2017). Naringenin ameliorates LPS-induced acute lung injury through its anti-oxidative and anti-inflammatory activity and by inhibition of the PI3K/AKT pathway. Exp. Ther. Med..

[159] Wang Q., Ou Y., Hu G., Wen C., Yue S., Chen C., Xu L., Xie J., Dai H., Xiao H. (2020). Naringenin attenuates non-alcoholic fatty liver disease by down-regulating the NLRP3/NF-κB pathway in mice. Br. J. Pharmacol..

[160] Kohno M., Musashi K., Ikeda H.O., Horibe T., Matsumoto A., Kawakami K. (2020). Oral administration of ferulic acid or ethyl ferulate attenuates retinal damage in sodium iodate-induced retinal degeneration mice. Sci. Rep..

[161] Narasimhan A., Chinnaiyan M., Karundevi B. (2015). Ferulic acid exerts its antidiabetic effect by modulating insulin-signalling molecules in the liver of high-fat diet and fructose-induced type-2 diabetic adult male rat. Appl. Physiol. Nutr. Metab. Physiol. Appl. Nutr. Metab..

[162] Wang J.M., Sheng Y.C., Ji L.L., Wang Z.T. (2014). Ferulic acid prevents liver injury and increases the anti-tumor effect of diosbulbin B in vivo. J. Zhejiang Univ. Sci. B.

[163] Hu Y., Li J., Chang A.K., Li Y., Tao X., Liu W., Wang Z., Su W., Li Z., Liang X. (2021). Screening and tissue distribution of protein tyrosine phosphatase 1B inhibitors in mice following oral administration of *Garcinia mangostana* L. ethanolic extract. Food Chem..

[164] Mahmoud A.M., Hussein O.E., Hozayen W.G., Bin-Jumah M., Abd El-Twab S.M. (2020). Ferulic acid prevents oxidative stress, inflammation, and liver injury via upregulation of Nrf2/HO-1 signaling in methotrexate-induced rats. Environ. Sci. Pollut. Res. Int..

[165] Wu J., Xue X., Fan G., Gu Y., Zhou F., Zheng Q., Liu R., Li Y., Ma B., Li S. (2021). Ferulic Acid Ameliorates Hepatic Inflammation and Fibrotic Liver Injury by Inhibiting PTP1B Activity and Subsequent Promoting AMPK Phosphorylation. Front. Pharmacol..

[166] Hawley S.A., Ford R.J., Smith B.K., Gowans G.J., Mancini S.J., Pitt R.D., Day E.A., Salt I.P., Steinberg G.R., Hardie D.G. (2016). The Na+/Glucose Cotransporter Inhibitor Canagliflozin Activates AMPK by Inhibiting Mitochondrial Function and Increasing Cellular AMP Levels. Diabetes.

[167] Craig S.A. (2004). Betaine in human nutrition. Am. J. Clin. Nutr..

[168] Holm P.I., Ueland P.M., Vollset S.E., Midttun Ø., Blom H.J., Keijzer M.B., den Heijer M. (2005). Betaine and folate status as cooperative determinants of plasma homocysteine in humans. Arterioscler. Thromb. Vasc. Biol..

[169] Kim S.K., Choi K.H., Kim Y.C. (2003). Effect of acute betaine administration on hepatic metabolism of S-amino acids in rats and mice. Biochem. Pharmacol..

[170] Go E.K., Jung K.J., Kim J.Y., Yu B.P., Chung H.Y. (2005). Betaine suppresses proinflammatory signaling during aging: The involvement of nuclear factor-kappaB via nuclear factor-inducing kinase/IkappaB kinase and mitogen-activated protein kinases. The journals of gerontology. Ser. A Biol. Sci. Med. Sci..

[171] Yi E.Y., Kim Y.J. (2012). Betaine inhibits in vitro and in vivo angiogenesis through suppression of the NF-κB and Akt signaling pathways. Int. J. Oncol..

[172] Ferrara N., Kerbel R.S. (2005). Angiogenesis as a therapeutic target. Nature.

[173] Muñoz-Chápuli R., Quesada A.R., Angel Medina M. (2004). Angiogenesis and signal transduction in endothelial cells. Cell. Mol. Life Sci..

[174] Blavier L., Lazaryev A., Dorey F., Shackleford G.M., DeClerck Y.A. (2006). Matrix metalloproteinases play an active role in Wnt1-induced mammary tumorigenesis. Cancer Res..

[175] Hussein R.M., Anwar M.M., Farghaly H.S., Kandeil M.A. (2020). Gallic acid and ferulic acid protect the liver from thioacetamide-induced fibrosis in rats via differential expression of miR-21, miR-30 and miR-200 and impact on TGF-β1/Smad3 signaling. Chem. Biol. Interact..

[176] Dos Santos A.N., de L. Nascimento T.R., Gondim B.L.C., Velo M.M.A.C., de A. Rêgo R.I., do C. Neto J.R., Machado J.R., da Silva M.V., de Araújo H.W.C., Fonseca M.G. (2020). Catechins as Model Bioactive Compounds for Biomedical Applications. Curr. Pharm. Des..

[177] Badolati N., Masselli R., Sommella E., Sagliocchi S., Di Minno A., Salviati E., Campiglia P., Dentice M., Tenore G.C., Stornaiuolo M. (2020). The Hepatoprotective Effect of Taurisolo, a Nutraceutical Enriched in Resveratrol and Polyphenols, Involves Activation of Mitochondrial Metabolism in Mice Liver. Antioxidants.

[178] Vestuto V., Amodio G., Pepe G., Basilicata M.G., Belvedere R., Napolitano E., Guarnieri D., Pagliara V., Paladino S., Rodriquez M. (2022). Cocoa Extract Provides Protection against 6-OHDA Toxicity in SH-SY5Y Dopaminergic Neurons by Targeting PERK. Biomedicines.

[179] Sugiyama H., Akazome Y., Shoji T., Yamaguchi A., Yasue M., Kanda T., Ohtake Y. (2007). Oligomeric procyanidins in apple polyphenol are main active components for inhibition of pancreatic lipase and triglyceride absorption. J. Agric. Food Chem..

[180] Hayakawa S., Saito K., Miyoshi N., Ohishi T., Oishi Y., Miyoshi M., Nakamura Y. (2016). Anti-Cancer Effects of Green Tea by Either Anti- or Pro-Oxidative Mechanisms. Asian Pac. J. Cancer Prev..

[181] Abe K., Ijiri M., Suzuki T., Taguchi K., Koyama Y., Isemura M. (2005). Green tea with a high catechin content suppresses inflammatory cytokine expression in the galactosamine-injured rat liver. Biomed. Res..

[182] Tipoe G.L., Leung T.M., Liong E.C., Lau T.Y., Fung M.L., Nanji A.A. (2010). Epigallocatechin-3-gallate (EGCG) reduces liver inflammation, oxidative stress and fibrosis in carbon tetrachloride (CCl4)-induced liver injury in mice. Toxicology.

[183] Ding Y., Sun X., Chen Y., Deng Y., Qian K. (2015). Epigallocatechin gallate attenuated non-alcoholic steatohepatitis induced by methionine- and choline-deficient diet. Eur. J. Pharmacol..

[184] Tang F.Y., Nguyen N., Meydani M. (2003). Green tea catechins inhibit VEGF-induced angiogenesis in vitro through suppression of VE-cadherin phosphorylation and inactivation of Akt molecule. Int. J. Cancer.

[185] Kenny T.P., Keen C.L., Jones P., Kung H.J., Schmitz H.H., Gershwin M.E. (2004). Cocoa procyanidins inhibit proliferation and angiogenic signals in human dermal microvascular endothelial cells following stimulation by low-level H_2_O_2_. Exp. Biol. Med..

[186] Zeng X., Feng Q., Zhao F., Sun C., Zhou T., Yang J., Zhan X. (2018). Puerarin inhibits TRPM3/miR-204 to promote MC3T3-E1 cells proliferation, differentiation and mineralization. Phytother. Res..

[187] Zheng P., Ji G., Ma Z., Liu T., Xin L., Wu H., Liang X., Liu J. (2009). Therapeutic effect of puerarin on non-alcoholic rat fatty liver by improving leptin signal transduction through JAK2/STAT3 pathways. Am. J. Chin. Med..

[188] Zhou J., Zhang N., Aldhahrani A., Soliman M.M., Zhang L., Zhou F. (2022). Puerarin ameliorates nonalcoholic fatty liver in rats by regulating hepatic lipid accumulation, oxidative stress, and inflammation. Front. Immunol..

[189] Park J., Min J.S., Kim B., Chae U.B., Yun J.W., Choi M.S., Kong I.K., Chang K.T., Lee D.S. (2015). Mitochondrial ROS govern the LPS-induced pro-inflammatory response in microglia cells by regulating MAPK and NF-κB pathways. Neurosci. Lett..

[190] Ma J.Q., Ding J., Zhao H., Liu C.M. (2014). Puerarin attenuates carbon tetrachloride-induced liver oxidative stress and hyperlipidaemia in mouse by JNK/c-Jun/CYP7A1 pathway. Basic Clin. Pharmacol. Toxicol..

[191] BedÊ T.P., Jesuz V.A., Souza V.R., Elias M.B., Oliveira F.L., Dias J.F., Teodoro A.J., Azeredo V.B. (2020). Effects of grape juice, red wine and resveratrol on liver parameters of rat submitted high-fat diet. An. Acad. Bras. Cienc..

[192] Yun H., Park S., Kim M.J., Yang W.K., Im D.U., Yang K.R., Hong J., Choe W., Kang I., Kim S.S. (2014). AMP-activated protein kinase mediates the antioxidant effects of resveratrol through regulation of the transcription factor FoxO1. FEBS J..

[193] Ahmed S.M., Luo L., Namani A., Wang X.J., Tang X. (2017). Nrf2 signaling pathway: Pivotal roles in inflammation. Biochim. Biophys. Acta Mol. Basis Dis..

[194] Bellezza I., Giambanco I., Minelli A., Donato R. (2018). Nrf2-Keap1 signaling in oxidative and reductive stress. Biochimica et biophysica acta. Mol. Cell Res..

[195] Di Pascoli M., Diví M., Rodríguez-Vilarrupla A., Rosado E., Gracia-Sancho J., Vilaseca M., Bosch J., García-Pagán J.C. (2013). Resveratrol improves intrahepatic endothelial dysfunction and reduces hepatic fibrosis and portal pressure in cirrhotic rats. J. Hepatol..

[196] de Oliveira C.M., Martins L.A.M., de Sousa A.C., Moraes K.D.S., Costa B.P., Vieira M.Q., Coelho B.P., Borojevic R., de Oliveira J.R., Guma F.C.R. (2021). Resveratrol increases the activation markers and changes the release of inflammatory cytokines of hepatic stellate cells. Mol. Cell. Biochem..

[197] Lee E.S., Shin M.O., Yoon S., Moon J.O. (2010). Resveratrol inhibits dimethylnitrosamine-induced hepatic fibrosis in rats. Arch. Pharmacal Res..

[198] Li B., Lu F., Wei X., Zhao R. (2008). Fucoidan: Structure and bioactivity. Molecules.

[199] Liu F., Wang J., Chang A.K., Liu B., Yang L., Li Q., Wang P., Zou X. (2012). Fucoidan extract derived from *Undaria pinnatifida* inhibits angiogenesis by human umbilical vein endothelial cells. Phytomed. Int. J. Phytother. Phytopharm..

[200] Abdollah M.R.A., Ali A.A., Elgohary H.H., Elmazar M.M. (2023). Antiangiogenic drugs in combination with seaweed fucoidan: A mechanistic in vitro and in vivo study exploring the VEGF receptor and its downstream signaling molecules in hepatic cancer. Front. Pharmacol..

[201] Lin K.I., Lin C.C., Kuo S.M., Lai J.C., Wang Y.Q., You H.L., Hsu M.L., Chen C.H., Shiu L.Y. (2018). Carnosic acid impedes cell growth and enhances anticancer effects of carmustine and lomustine in melanoma. Biosci. Rep..

[202] Johnson J.J. (2011). Carnosol: A promising anti-cancer and anti-inflammatory agent. Cancer Lett..

[203] López-Jiménez A., García-Caballero M., Medina M.Á., Quesada A.R. (2013). Anti-angiogenic properties of carnosol and carnosic acid, two major dietary compounds from rosemary. Eur. J. Nutr..

